# Asymmetry Indices for Analysis and Prediction of Replication Origins in Eukaryotic Genomes

**DOI:** 10.1371/journal.pone.0045050

**Published:** 2012-09-27

**Authors:** Marie-Claude Marsolier-Kergoat

**Affiliations:** Institut de Biologie et de Technologies de Saclay, CEA/Saclay, Gif-sur-Yvette, France; The University of Nottingham, United Kingdom

## Abstract

DNA replication was recently shown to induce the formation of compositional skews in the genomes of the yeasts *Saccharomyces cerevisiae* and *Kluyveromyces lactis*. In this work, I have characterized further GC and TA skew variations in the vicinity of *S. cerevisiae* replication origins and termination sites, and defined asymmetry indices for origin analysis and prediction. The presence of skew jumps at some termination sites in the *S. cerevisiae* genome was established. The majority of *S. cerevisiae* replication origins are marked by an oriented consensus sequence called ACS, but no evidence could be found for asymmetric origin firing that would be linked to ACS orientation. Asymmetry indices related to GC and TA skews were defined, and a global asymmetry index *I_GC,TA_* was described. *I_GC,TA_* was found to strongly correlate with origin efficiency in *S. cerevisiae* and to allow the determination of sets of intergenes significantly enriched in origin loci. The generalized use of asymmetry indices for origin prediction in naive genomes implies the determination of the direction of the skews, *i.e.* the identification of which strand, leading or lagging, is enriched in G and which one is enriched in T. Recent work indicates that in *Candida albicans* and in several related species, centromeres contain early and efficient replication origins. It has been proposed that the skew jumps observed at these positions would reflect the activity of these origins, thus allowing to determine the direction of the skews in these genomes. However, I show here that the skew jumps at *C. albicans* centromeres are not related to replication and that replication-associated GC and TA skews in *C. albicans* have in fact the opposite directions of what was proposed.

## Introduction

In 1995, Lobry and Sueoka demonstrated that, in the absence of selective or mutational differences between the two complementary strands of DNA, the composition of a single DNA strand at equilibrium should be such that *G* = *C* and *T* = *A*, with *A*, *C*, *G* and *T* representing the frequencies of adenine, cytosine, guanine and thymine, respectively [1], [2]. Several functions and processes, in particular protein coding and replication, induce asymmetries between complementary DNA strands. The resulting departure from the intrastrand parity rules *G* = *C* and *T* = *A* can be quantified by computing the GC and TA skews as the ratios (*G*−*C*)/(*G*+*C*) and (*T*−*A*)/(*T*+*A*), respectively.

Replication induces differences between a leading and a lagging strand, which are synthesized continuously and discontinuously, respectively. The leading and the lagging strands were shown to have different substitution rates in several systems (*e.g.* [3], [4]), which could be due to differences in the mutational spectrum of the leading- and the lagging-strand polymerases, to differential mismatch repair of leading-strand versus lagging-strand replication errors or to the difference in the frequency with which the leading and the lagging strands are in single-strand DNA conformation (for reviews, see [3], [5]).

The different substitution rates could be expected to lead to different values of GC and TA skews for the leading and the lagging strands, which would mark replication origins and termination sites as positions where these values abruptly change. Lobry found in 1996 that that was indeed the case: he showed that the values of GC and TA skews calculated along large fragments of several eubacterial genomes exhibit a sharp jump at the position of the replication origin, thus demonstrating the possibility of origin prediction through analysis of compositional biases [6]. These observations were soon generalized [7], [8] and the analysis of GC and TA skews is now a standard method to predict replication origins in eubacteria [9], [10].

Theoretically, the termination sites of eubacterial chromosomes should also appear as skew jumps, with an inverted orientation compared to origins. One sharp skew jump is often detected in the termination region of eubacterial genomes, suggesting that the fork convergence points are precisely located at this site, and not at the multiple unidirectional replication pause (*Ter*) sites that may span a large portion of the genome (about half the *Escherichia coli* chromosome for example) [11]. The experimental detection in *E. coli* of replication fork arrests and of converging forks at some *Ter* sites (but not at the site of the skew jump) contradicts the hypothesis that the site of the skew jump would be currently used as a termination site [12], but could be explained by the recent appearance of a *Ter* site-based termination mechanism which would have superseded a previous mechanism using the position of the skew jump as termination site. Alternatively, the skew jump in the termination region could be due to compositional biases unrelated to replication and possibly linked to chromosome recombination or segregation. The possibility to predict termination sites in eubacterial genomes through analysis of compositional skews thus remains unclear.

In contrast to eubacteria with their one unique origin per chromosome, replication is more complex in eukaryotes where stochasticity plays a large part [13]. Multiple potential replication origins exist in eukaryotic genomes and, during a given S phase, only a minority of them fire while most are passively replicated (*e.g.* [14]). Since replication origins do not fire systematically in all S phases, a given sequence can be synthesized as a leading strand during some replication cycles and as a lagging strand in other cycles, thus reducing replication-related GC and TA skews.

Nevertheless, the existence of replication-associated GC and TA skews was recently proposed for the human genome [15], [16], [17] and for the genomes of two yeast species, *Saccharomyces cerevisiae* and *Kluyveromyces lactis* [18], [19]. In the human genome, a sharp upward jump of the total skew *S*, defined as (*G*−*C*)/(*G*+*C*)+(*T*−*A*)/(*T*+*A*), was first detected at some experimentally-determined replication origins [17]. This led to an origin-prediction method, which provided a set of ∼1,500 upward skew jumps or putative origins. The skew *S* calculated for intergenic regions (in order to eliminate transcription-associated skews) was found to decrease linearly along the interorigin intervals and no downward jumps that would have reflected the presence of precisely positioned termination sites were observed between two adjacent predicted origins [16], [17]. About 56% of these putative origins were subsequently found to be located at a distance less than 100 kb from a replication initiation zone in at least one of six different cell types, which indicates that upward jump positions correspond probably to replication origins active in germline cells [15]. However, these putative origins represent only a fraction of the 30,000 to 100,000 origins expected in human germline cells, probably the earliest and the most efficient ones.

We and others have recently demonstrated in *S. cerevisiae* and in *K. lactis* the existence of replication-associated GC and TA skews and the enrichment of the leading strand in adenines and cytosines [18], [19]. In this work, I have first sought to further characterize skew variations around replication origins and termination sites in *S. cerevisiae*, where they are the most documented. Asymmetry indices linked to GC and TA skews were subsequently defined and a global asymmetry index *I_GC,TA_* was described. *I_GC,TA_* was found to strongly correlate with origin efficiency and to allow the definition of sets of intergenes significantly enriched in origin loci in *S. cerevisiae*. The generalized use of asymmetry indices for origin prediction in naive genomes implies the determination of the direction of the skews. In *Candida albicans* and in related species, the centromeric regions contain early and efficient replication origins. It has been proposed that the skew jumps observed at these positions would reflect the activity of these origins, thus allowing to determine the direction of the skews in these genomes [20]. However, I show here that the skew jumps at *C. albicans* centromeres are not related to replication and that replication-associated GC and TA skews in *C. albicans* have in fact the opposite directions of what was previously suggested.

## Materials and Methods

### Sequence Data

*S. cerevisiae* sequences were downloaded from the GenBank web page ftp://ftp.ncbi.nih.gov/genomes/Fungi/Saccharomyces_cerevisiae_uid128/(version of July 2011) and *C. albicans* sequences (SC5314 assembly 21, version of November 2011) were obtained from the *Candida* Genome Database (http://www.candidagenome.org/download/sequence/C_albicans_SC5314/Assembly21/current/).

For all analyses of *S. cerevisiae* and *C. albicans* coding sequences, only the 5775 and 6030 open reading frames (ORFs) annotated as verified or uncharacterized by the *Saccharomyces* Genome Database (http://www.yeastgenome.org/) and the *Candida* Genome Database, respectively, were taken into account. Intergenes were defined as the intervals between the translated regions of verified or uncharacterized genes.

### Replication Origins

For *S. cerevisiae*, the OriDB database (http://www.oridb.org/index.php, [21]) was used as reference for the features of confirmed, likely, and dubious origins. In particular, replication time for confirmed origins was taken as the average of the relative replication times determined by two whole-genome timing studies [22], [23], as indicated by OriDB. To analyze the correlation between asymmetry index and origin efficiency, I considered the parameter “observed efficiency” of the origins listed in Supplementary Table 1 of reference [24], that colocalize with origins annotated as confirmed in OriDB. The locus of an origin from Supplementary Table 1 was considered to colocalize with the locus of a confirmed origin from OriDB if they overlap or if their midpoints are located less than 10 kb apart (the median distance between the midpoints of two origins considered as colocalized was ∼ 1 kb).

Origin positions for *C. albicans* were extracted from the replication timing profiles determined in [20]. The original data (GSE17963_final_data.txt) corresponding to one value of replication time every 50 bp were averaged using non-overlapping windows of 500 bp to derive a restricted dataset of positions *x* (in bp) with replication times *rep(x)*. All positions *x* such that *rep(x*−*500)*<*rep(x)* and *rep(x)*>*rep(x+500)*, corresponding to peaks in the replication profiles, were considered as origin positions. A total number of 128 origins were thus identified. It has to be stressed that the number and positions of peaks in replication timing profiles only approximate the number and positions of replication origins since only the earliest and most efficient origins are detected.

### Analysis of GC and TA Skews

GC and TA skews were determined as previously described [19], using only coding (and not template) sequences when studying ORFs. When the data from all interorigin intervals are pooled, for the analysis of *C. albicans*, fractional positions are used to account for the variability of interorigin interval length.

The codon adaptation index (CAI) values for *C. albicans* genes were computed as described in [19], using the algorithm defined in [25]. The most biased reference set of genes contains almost exclusively genes encoding ribosomal proteins, elongation factors, glycolytic and heat-shock proteins, which indicates that expression level plays an important part in biased codon usage in *C. albicans*.

### Receiver Operator Characteristic (ROC) Curves

To evaluate the ability of asymmetry indices to discriminate between origin-lacking and origin-containing intergenes in *S. cerevisiae*, a set of 274 origins annotated as confirmed and chromosomically active by OriDB was defined. The analysis was then restricted to the 263 origin loci that overlap with at least one intergene (given the start and end points indicated by OriDB, 256 origin loci overlap with one intergene and 7 origin loci overlap with two intergenes). All intergenes overlapping with an origin locus were considered as containing an origin. The values of asymmetry indices for intergenes were computed either at a unique position corresponding to intergene midpoint, when intergene length was less than 1.5 kb, or at several positions 1-kb distant from each other, when the intergene was longer. When several values of an asymmetry index were computed for a long intergene, only the highest one was taken into account.

### Computational and Statistical Analyses

Data sets were produced and analyzed with custom Python scripts. Statistical analyses were performed with the R environment [26]. When Wilcoxon rank-sum tests were used to evaluate the significance of differences in the GC and TA skew curves between the right and the left sides of origins or the significance of the peaks and troughs observed for asymmetry index curves, the values corresponding to a given origin or termination site were paired. The variations with interorigin position of the GC and TA skews for *C. albicans* were analyzed using generalized linear models as described in [19].

## Results and Discussion

### No Evidence for Asymmetric Origin Firing Linked to ACS Orientation in *S. cerevisiae*

The large majority of replication origins in *S. cerevisiae* are characterized by the presence of an asymmetric 11-bp motif named the ARS (Autonomously Replicating Sequence) Consensus Sequence or ACS (5′-[A/T]TTTAT[A/G]TTT[A/T]-3′), which is essential for their function [27], [28], [29]. It is currently unknown whether ACS asymmetry is translated into the assembly of two symmetrical replication forks. In *E. coli*, the replication origin *oriC* is also asymmetric and the leftward fork is systematically ahead compared to the rightward fork, with a strain-specific mean offset size of ∼ 40 to ∼ 150 kb which is established at or just after initiation [30]. Similarly, the large variability in the location of fork convergence points observed in the case of a single-origin plasmid replicated in *S. cerevisiae* suggests the possibility of asynchronous departures of the two forks starting from an origin and of differences in the rates of fork progression [31].

I looked for evidence of asymmetric origin firing in *S. cerevisiae* by analyzing GC and TA skew variations around origins oriented by their ACS. Since mutational processes are analyzed, only sequences undergoing the weakest selective pressure can valuably be taken into account. For the *S. cerevisiae* genome, in which introns are scarce, the analysis was limited to third codon positions and to intergenes. Under certain conditions, differences in the behaviors of the forks departing on the right and left sides of oriented origins could lead to differences in the variations of GC and TA skews on the two sides. For example, if the forks departing on the right side of origins started systematically earlier than the forks departing on the left side, the GC and TA skews would be expected to decrease with distance from the origin on the left side more rapidly than they increase on the right side (because fork convergence points would be located closer to the origin on the left side than on the right side).

**Figure 1:**
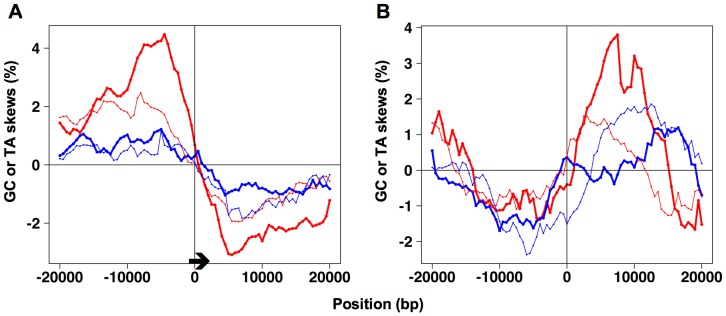
GC and TA skew jumps at replication origins (A) and at termination sites (B) in *Saccharomyces cerevisiae*. Average values of GC and TA skews computed for third codon positions (thick, red line and thick, blue line, respectively) and for intergenes (thin, red line and thin, blue line, respectively) were calculated every 500 bp over a sliding window of 10 kb. Position 0 corresponds to the position of the extended ACS sequence 5′-(T/A)(T/G)TTTAT(G/A)TTT(T/A)(G/C)(T/G)T-3′ [32] for origins (A) and to the midpoint of termination regions (B). Average GC and TA skew values related to protein coding were substracted from the total GC and TA skew values calculated for third codon positions, so as to plot on the same graph the skew curves corresponding to intergenes and to third codon positions. ACS orientation is marked by an arrow in (A).

The average variations of GC and TA skews were computed over a 10-kb sliding window around 193 confirmed, chromosomically active replication origins, whose ACS position and orientation have been precisely determined by Nieduszynski and collaborators [21], [32]. The data were processed so as to display skew variations around all origins oriented in the same direction. As shown in Figure 1A, the ACS (located at position 0) is marked by skew jumps but no obvious difference can be observed in the way average GC and TA skews decrease and increase with distance from the ACS on the left and right sides, respectively. For the GC skews and for the TA skew computed for third codon positions, Wilcoxon rank-sum tests confirmed the absence of significant difference between the absolute values of the skews calculated at positions located at the same distance from the ACS on the right and left sides. The absolute values of the TA skew computed for intergenes are significantly different (Wilcoxon rank-sum test, *P* value <0.01) on the right and left sides of the ACS at distances of 7000, 7500, 11,500 and 12,000 bp. Since only a few points show significant differences, and only for the TA skew computed for intergenes (whose values show the weakest correlation with the interorigin position, when compared to the other skews [19]), I conclude that no evidence for asymmetric origin firing that would be linked to ACS orientation can be detected.

Figure 1A also shows two features supporting the hypothesis of replication initiating systematically close to the position of the ACS. If we consider the GC skew computed for third codon positions (whose values show the strongest correlation with interorigin position [19] and the largest variations), we see that the extrema of the curve are located almost exactly at positions −5000 and 5000 bp (which is what is expected given that the skews are computed over 10 kb) and that the curve decreases linearly between positions −5000 and 5000 bp. The sharpness of the peak and trough suggests that polymerization mostly starts close to the ACS because if replication initiated at many origin loci systematically a few kb away from the ACS, either on the right or on the left side, the average skew curve would exhibit rounded peak and trough. The other skew curves do not show exactly this ideal behavior but it can be noted that in all cases the zero-intersection point is at or close to the precise location of the ACS, in keeping with the hypothesis that polymerization starts on average close to this position.

### Some Termination Sites are Marked by Skew Jumps in *S. cerevisiae*

Termination sites have been much less characterized than origins in *S. cerevisiae*: only 71 termination loci were identified as 2-to-10 kb zones in a recent study [33]. Average values of GC and TA skews were computed over 10-kb windows in the vicinity of these termination regions with position 0 corresponding to their midpoints. As shown in Figure 1B, both GC and TA skews exhibit upward jumps around the average midpoint of termination sites. However, the skew curves are obviously less symmetric than in Figure 1A and the extrema of the curve corresponding to the GC skew of third codon positions are not located exactly at positions −5000 and 5000 bp, in keeping with the lack of precise positioning of termination sites.

Skew jumps corresponding to termination sites have not been observed in the human genome [16], [17], which suggests large variations in the position of the fork convergence point between two adjacent origins. In contrast, the presence of average skew jumps in termination regions in yeast indicates, at least in some cases, a fixed position for termination sites (although they can extend over several kb), which is consistent with the observation that in *S. cerevisiae* clusters of topoisomerase II (required for optimal replication termination) are established at termination sites in early S-phase, independently of origin firing [33].

### Characterizing Chromosomal Loci by Asymmetry Indices

The use of a locus as a replication origin or as a termination site induces differences between the replication-associated skews of the sequences located on its left and right sides. The amplitude of these differences can be visualized as the height of skew jumps at these loci (Figure 1). I propose to quantify these differences in replication-associated skews by asymmetry indices which can be computed for any position and reflect the activity of nearby origin or termination sites. I will first detail the computation of these indices and then analyze their relationships with other features of origin loci.

**Figure 2:**
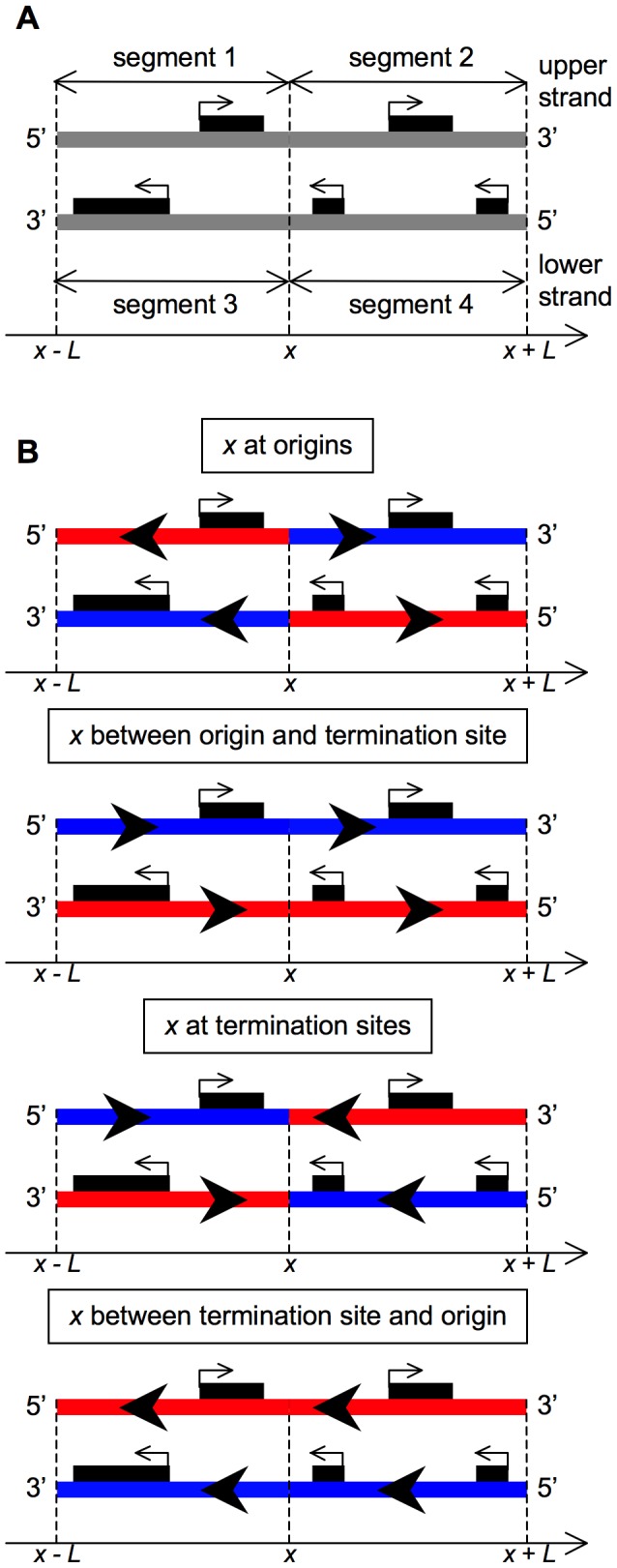
Schematics illustrating the computation of asymmetry indices. (A) Definition of the four strand segments. (B) Four simple cases are shown for different positions *x*. Segments synthesized as leading and lagging strands are shown as blue and red lines, respectively. The small, black rectangles represent coding sequences with their transcriptional orientation given by the associated arrows. The large, black arrowheads indicate the orientation of the replication forks.

Asymmetry indices are computed separately for third codon positions and for intergenes. Analysis of third codon positions is complicated by the presence of coding sequence-associated skews (induced by transcription-related mutational processes or selective pressures on codon usage), which have opposite signs in coding and template sequences. In order to eliminate coding sequence-associated skews and define an index that best quantifies the difference in replication-related skews between the left and right sides of a given position, I analyze only coding sequences (the consequences of mixing coding and template sequences are discussed afterwards) and proceed as follows. For a given position *x* on a chromosome and a window length *L*, four strand segments are considered: segment 1 corresponds to the upper strand segment between positions *x-L* and *x*, (*i.e.* to the interval [*x-L*,*x*)), segment 2 corresponds to the upper strand interval (*x*,*x+L*], and segments 3 and 4, to the lower strand intervals [*x*−*L*,*x*) and (*x*,*x+L*], respectively (Figure 2A). Let *A_i_*, *C_i_*, *G_i_* and *T_i_* represent, respectively, the numbers of adenines, cytosines, guanines and thymines at third codon positions in the coding sequences located on the strand segment *i*. A GC asymmetry index, *I_GC,cod_*, and a TA asymmetry index, *I_TA,cod_*, related to the GC and the TA skews, respectively, can be computed:
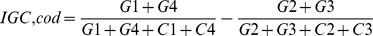


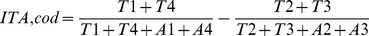



These indices correspond to the difference between the skews of segments 1 and 4, and those of segments 2 and 3. As illustrated in Figure 2B, when *x* corresponds to efficient origins or termination sites, segments 1 and 4 should exhibit the same skews since they are both synthesized either as lagging (if *x* is at an origin) or leading (if *x* is at a termination site) strand. In these cases, segments 2 and 3 should also present similar skews, different from the skews of segments 1 and 4, so that the values of the asymmetry indices should be significantly non null. In contrast, when *x* is far from origins and termination sites, the skews of segments 1 and 4 should differ to the same extent as the skews of segments 2 and 3, so that the asymmetry indices should not be significantly different from zero. For simplicity, GC and TA skews are computed in this case as *G*/(*G*+*C*) and *T*/(*T*+*A*), instead of (*G*−*C*)/(*G*+*C*) and (*T*−*A*)/(*T*+*A*), respectively, since the formulae are obviously related:
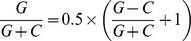



Another possibility for the definition of asymmetry indices for third codon positions would be to compute the skew difference between the mix of coding and template sequences located on segment 1 and those located on segment 2. However, in this case, the skew difference between segments 1 and 2 would reflect differences in the proportions of coding and template sequences in these segments. Let’s suppose as an extreme example that segments 1 and 2 would harbor only coding sequences and only template sequences, respectively. The computation of the skew difference for third codon positions between segments 1 and 2 would integrate the difference of coding sequence-associated skew between coding and template sequences, which could strongly distort the estimation of replication-associated skews. In contrast, analyzing skew differences between coding sequences on the upper and lower strands eliminates coding sequence-associated skews and provides a more accurate measure of the difference in the replication-associated skews between the left and right sides of a given position.

Similar indices can be computed for intergenes in a simpler way since the complementary strands of an intergene do not need to be distinguished. Only segments 1 and 2 are considered and the GC and TA asymmetry indices *I_GC,int_* and *I_TA,int_*, are computed as:
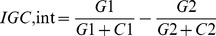


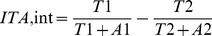
with *A_i_*, *C_i_*, *G_i_* and *T_i_* representing, respectively, the numbers of adenines, cytosines, guanines and thymines of the intergenes located on the strand segment *i*.

**Figure 3:**
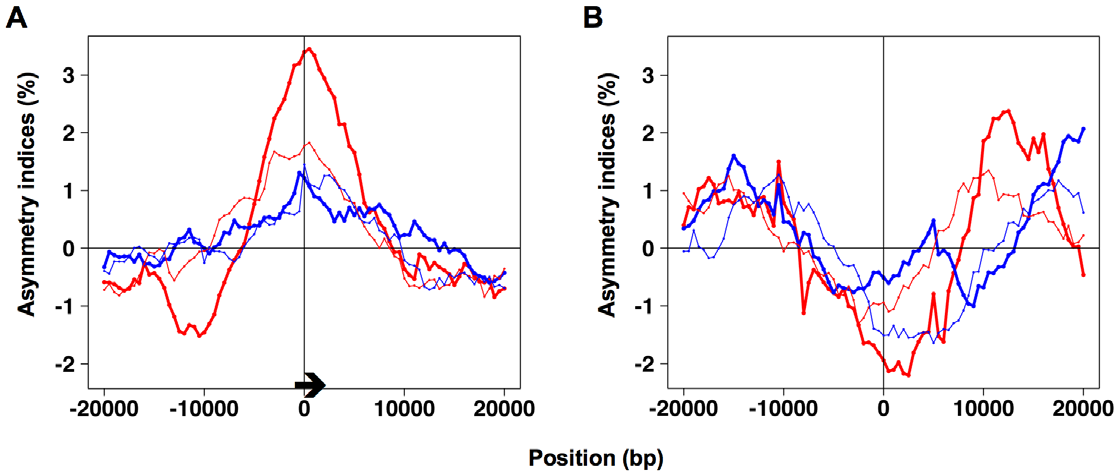
Variations of asymmetry indices around replication origins (A) and termination sites (B) in *Saccharomyces cerevisiae*. The average values of *I_GC,cod_* (thick, red line), *I_TA,cod_* (thick, blue line), *I_GC,int_* (thin, red line) and *I_TA,int_* (thin, blue line) were computed every 500 bp using a window length *L* = 10 kb. Position 0 corresponds to the position of the extended ACS sequence 5′-(T/A)(T/G)TTTAT(G/A)TTT(T/A)(G/C)(T/G)T-3′ [32] for origins (A) and to the midpoint of termination regions (B). All origins were oriented according to their ACS, as indicated by the arrow in (A).

The average values of asymmetry indices were computed around the 193 confirmed origins previously analyzed (with well-characterized ACS position and orientation) [21], [32] and around the 71 termination sites described in [33]. The curves representing the indices exhibit globally the expected peaks and troughs, respectively, around the ACS of replication origins and the midpoints of termination sites, taken as position 0 (Figure 3). However, these variations are more or less marked depending on the indices, and their significance was quantified by comparing the indices values at position 0 with their values at positions −10,000, −5000, 5000 or 10,000 bp, using Wilcoxon rank-sum tests. Around replication origins, the values of all indices at position 0 are significantly higher than their values at positions −10,000, −5000, 5000 or 10,000 bp (*P* values <0.01 for *I_GC,cod_*, *I_GC,int_* and *I_TA,int_*, *P* values <0.05 for *I_TA,cod_*). Around termination sites, the values of *I_GC,cod_* at position 0 are significantly lower than its values at positions −10,000 or 10,000 (*P* values <0.05), the values of *I_GC,int_* at position 0 are significantly lower than its values at position 10,000 bp (*P* value <0.01) and the values of *I_TA,int_* at position 0 are significantly lower than its values at positions −10,000, −5000 or 10,000 bp (*P* values <0.05). The other indices values at positions −10,000, −5000, 5000 or 10,000 bp are not significantly different from the corresponding values at position 0, which can be at least partly explained by the lack of precise positioning of the termination sites. In the following we will focus on the asymmetry indices of replication origins.

### Origin Efficiency Correlates with Global Asymmetry Index in *S. cerevisiae*

**Figure 4:**
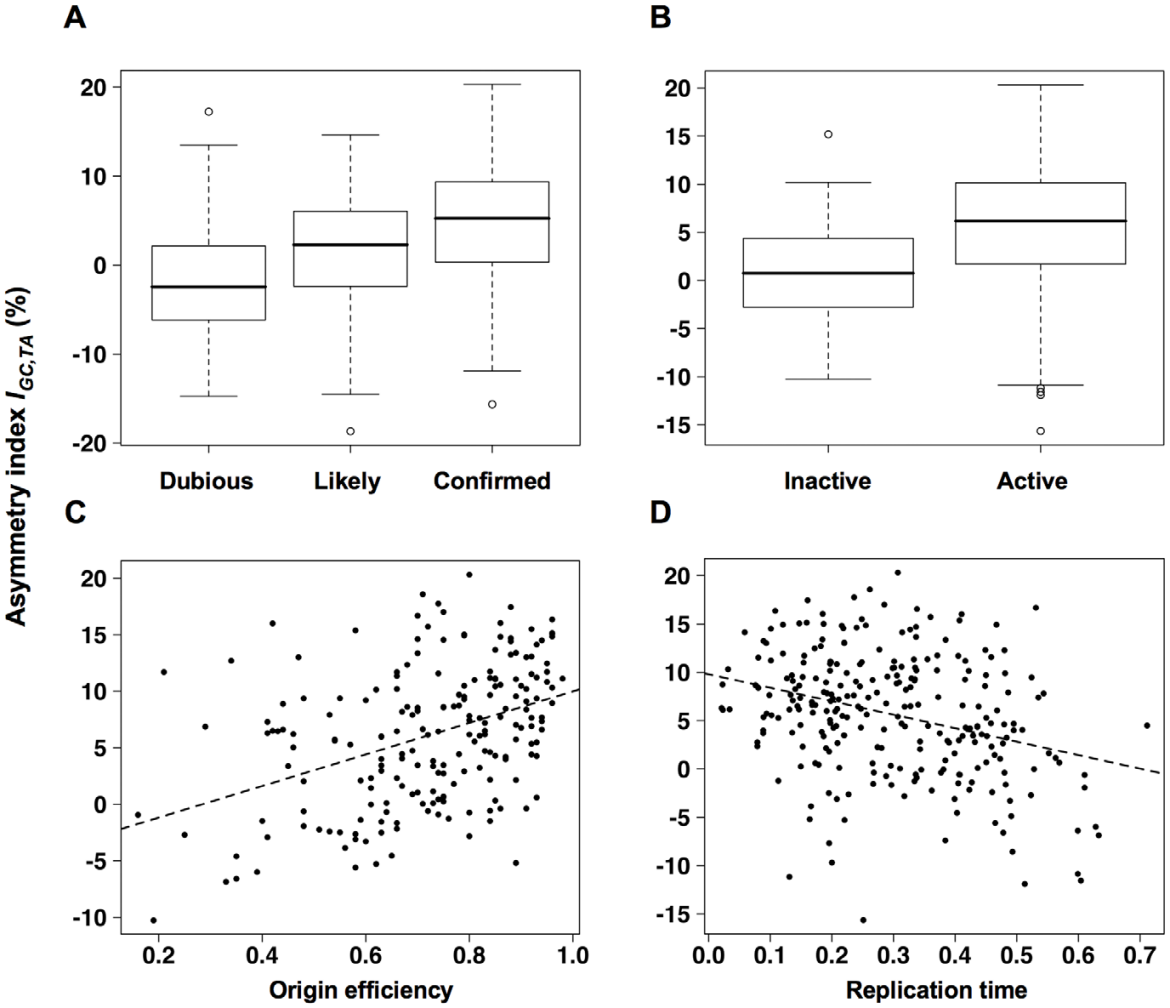
Origin efficiency correlates with the global asymmetry index *I_GC,TA_* in *Saccharomyces cerevisiae*. (A,B) Box plots displaying the differences in *I_GC,TA_* values between replication origins annotated as confirmed, likely, and dubious (A) or as chromosomically active and inactive (B). The horizontal lines show the median values. The bottoms and tops of the boxes show the 25th and the 75th percentiles, respectively. (C,D) *I_GC,TA_* values are plotted as a function of origin efficiency and average replication time, respectively. The dashed lines correspond to least square fits.

To simplify correlation analysis, a global asymmetry index *I_GC,TA_* was defined as the unweighted sum *I_GC,cod_*+*I_TA,cod_*+*I_GC,int_*+*I_TA,int_*, computed for a window length *L* = 20 kb. The choice of *L* was motivated by the fact that the mean interorigin distance is ∼ 40 kb in *S. cerevisiae*, considering the 274 origins annotated as confirmed and chromosomically active in the OriDB database [21]. Moreover, as shown below, *L* = 20 kb allows the best discrimination of origin-containing from origin-lacking intergenes. The relevance of *I_GC,TA_* for origin analysis was first tested by comparing three sets of replication origins that have been defined as confirmed, likely, and dubious in OriDB. The values of *I_GC,TA_* were computed for the position corresponding to the midpoint of origin loci, as defined in OriDB. The values of *I_GC,TA_* for confirmed origins were found significantly higher than those of likely origins (Wilcoxon rank-sum test, *P* value = 3×10^−7^), which are in turn significantly higher than those of dubious origins (Wilcoxon rank-sum test, *P* value = 4×10^−8^), thus demonstrating the discriminative power of *I_GC,TA_* (Figure 4A). The remainder of the analysis is restricted to confirmed origins.

For replication origins, *I_GC,TA_* is an increasing function of the number of S phases in which they have fired. Origin efficiency is defined as the frequency with which an origin fires during S phase, as opposed to being passively replicated. The number of S phases in which an origin has fired corresponds to the integration over time of the origin efficiency between the time of its appearance up to the time when the locus ceases to be used as a replication origin. *I_GC,TA_* is thus expected to correlate positively with both origin efficiency and age.

Among the 309 confirmed origins for which *I_GC,TA_* values could be computed (*i.e.* whose midpoints are located at least 20 kb away from telomeres), 50 are considered to be chromosomically inactive for replication initiation, as they were identified by neither of two whole-genome timing studies [22], [23]. The values of *I_GC,TA_* for the chromosomically inactive origins were found significantly lower than those of the active ones (Wilcoxon rank-sum test, *P* value = 7×10^−8^, Figure 4B). Regarding chromosomically active origins, efficiency has been directly measured only in a few cases [14], but Bechhoefer and collaborators recently developed an analytical model incorporating probabilistic initiation of origins and passive replication that allows to extract origin efficiency from replication time course microarray data [24]. *I_GC,TA_* was found to strongly correlate with the calculated origin efficiency (Spearman correlation coefficient *ρ* = 0.42, *P* value = 8×10^−10^, *N* = 202, Figure 4C). As expected from the anticorrelation between origin efficiency and replication time [24], *I_GC,TA_* is also negatively correlated with replication time (*ρ = *−0.29, *P* value = 2×10^−6^, *N* = 259, Figure 4D). These results validate *I_GC,TA_* as a correlate of origin efficiency that can be used to compare sets of origins.

### Asymmetry Indices as Predictors of Replication Origins

Having established the correlation between *I_GC,TA_* and origin efficiency, I examined the performance of asymmetry indices as predictors of replication origins. Wavelet-based methods have been successfully developed for skew analysis in mammalian genomes (for a review, see [34]), but so far the most efficient approach for origin prediction in small, compact genomes is to compute GC and TA skews in sliding windows over the whole length of chromosomes and to inspect jumps, or to examine the extrema of cumulative skew curves (for a methodological review, see [35]). Taking into account the variations of GC and TA skews over large chromosomal fragments is well adapted to the prediction of unique replication origins in eubacterial genomes, as in these cases the positions of skew jumps are often separated by large regions with globally uniform skew values [6], [7], [8]. However, this type of approach (detrended DNA walks) proved unable to detect replication-related skews in *S. cerevisiae* [18], [19], [36]. Asymmetry indices seem therefore more suited to origin prediction in compact, eukaryotic genomes for two reasons. First, asymmetry indices do not mix coding and template sequences when analyzing third codon positions. This eliminates the distortion of replication-associated skew estimation due to coding sequence-associated skews, whereas this source of distortion is present in the computation of GC and TA skews in sliding windows over chromosome fragments. Second, in genomes where the mean interorigin distance is ∼ 40 kb, only the skews of the sequences immediately adjacent on both sides of a given locus are relevant for estimating the probability for this locus to be used as an origin.

The predictive approach using asymmetry indices can be made more powerful by using additional information about the preferential localization of origins. Replication origins are almost exclusively located in intergenic regions in the three yeast species (*S. cerevisiae*, *Schizosaccharomyces pombe* and *K. lactis*) for which they have been precisely mapped. A simple strategy for origin prediction in yeast genomes thus consists in estimating the probability of all intergenes to be used as an origin by computing their asymmetry indices. The performance of this approach was assessed for *S. cerevisiae*.

**Figure 5:**
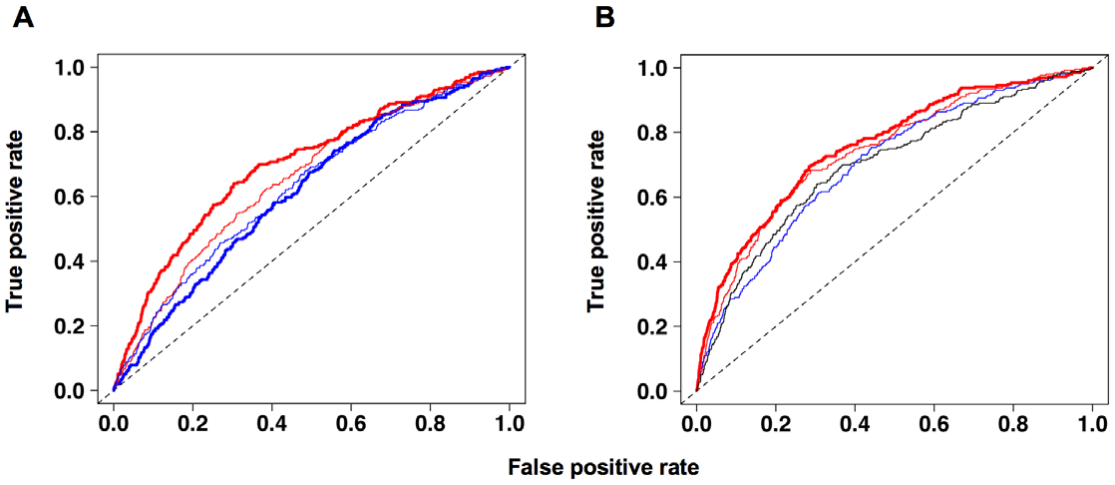
ROC curves displaying the discriminative power of the asymmetry indices *I_GC,cod_*, *I_TA,cod_*, *I_GC,int_* and *I_TA,int_*. (A) Intergenes were classified according to their values of *I_GC,cod_* (thick, red line), *I_TA,cod_* (thick, blue line), *I_GC,int_* (thin, red line) and *I_TA,int_* (thin, blue line). (B) Intergenes were classified according to their values of *I_GC,cod_*+*I_TA,cod_* (thin, red line), *I_GC,int_*+*I_TA,int_* (thin, blue line) and *I_GC,TA_* (thick, red line). The ROC curve corresponding to *I_GC,cod_* (thin, black line) is shown for comparison.

The values of the indices *I_GC,cod_*, *I_TA,cod_*, *I_GC,int_* and *I_TA,int_* were computed using a window length *L* = 20 kb for all *S. cerevisiae* intergenes (see Materials and Methods). Intergenes were then classified according to their values of *I_GC,cod_*, *I_TA,cod_*, *I_GC,int_* and *I_TA,int_* either taken independently (Figure 5A) or in combination (Figure 5B). The discriminative power of the various indices and of their combinations is presented as receiver operator characteristic (ROC) curves, with the *y*-axis representing the sensitivity of the assay (true-positive rate) as a function of 1-specificity (false-positive rate). As could have been expected from its strong variations around origins (Figure 3A), *I_GC,cod_* had a higher discriminative power than *I_TA,cod_*, *I_GC,int_* and *I_TA,int_* considered individually (Figure 5A, the values of the areas under curves (AUC) are equal to 0.69, 0.61, 0.65 and 0.62, respectively, for *I_GC,cod_*, *I_TA,cod_*, *I_GC,int_* and *I_TA,int_*). Adding the values of *I_GC,cod_* and *I_TA,cod_* or of *I_GC,int_* and *I_TA,int_* increases the accuracy of the predictions compared to the use of individual predictor variables (AUC values equal to 0.73 and 0.69, respectively), and the best performance was obtained with the global asymmetry index *I_GC,TA_* (Figure 5B, AUC value = 0.75). In that latter case, 30, 50 and 63 origin-containing intergenes, respectively, are found among the 100, 200 and 300 intergenes with the highest *I_GC,TA_* values. Since origin-containing intergenes represent 4.6% of all intergenes analyzed, the random selection of 100, 200 and 300 intergenes yields on average 5, 9 and 14 origin-containing intergenes, respectively, which is significantly different from the numbers obtained using *I_GC,TA_* values (binomial proportion test, *P* values equal to 8×10^−6^, 2×10^−8^ and 5×10^−9^, respectively). The same test was carried out using a window length *L* of 5, 10, 15, 25 and 30 kb for computing *I_GC,TA_*, with globally similar, although lower, performances (data not shown).

**Figure 6:**
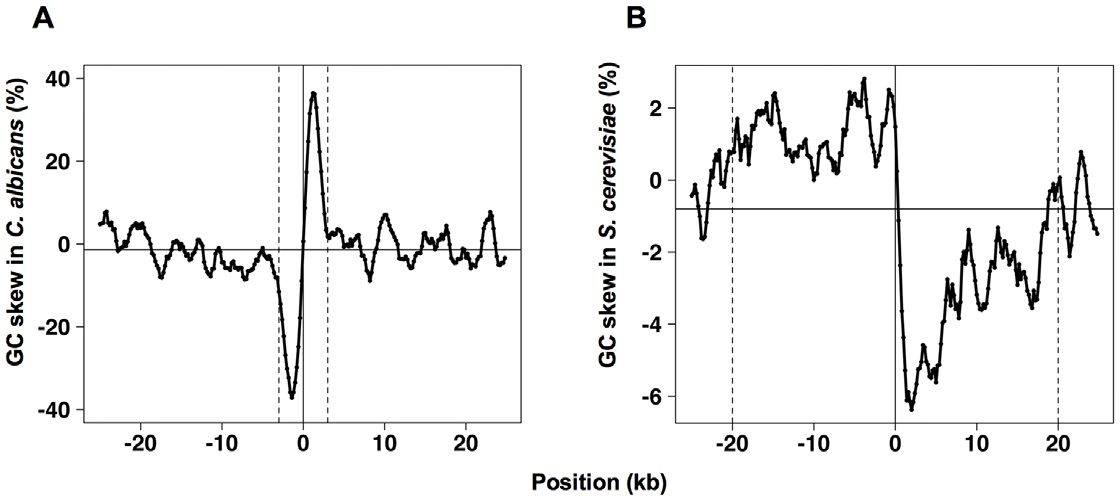
Variations of GC skew around *Candida albicans* centromeres (A) and around *Saccharomyces cerevisiae* replication origins (B). (A) Average values of GC skew were computed every 200 bp using sliding windows of 1.5 kb and taking into account all sequences, as described in [20]. (B) Average GC skew values were computed every 200 bp using sliding windows of 1.5 kb and taking into account third codon positions. The horizontal lines correspond to the mean GC skew values, averaged over all positions. The dashed, vertical lines mark positions −3 and 3 (kb) for *C. albicans* and positions −20 and 20 (kb) for *S. cerevisiae*.

**Figure 7:**
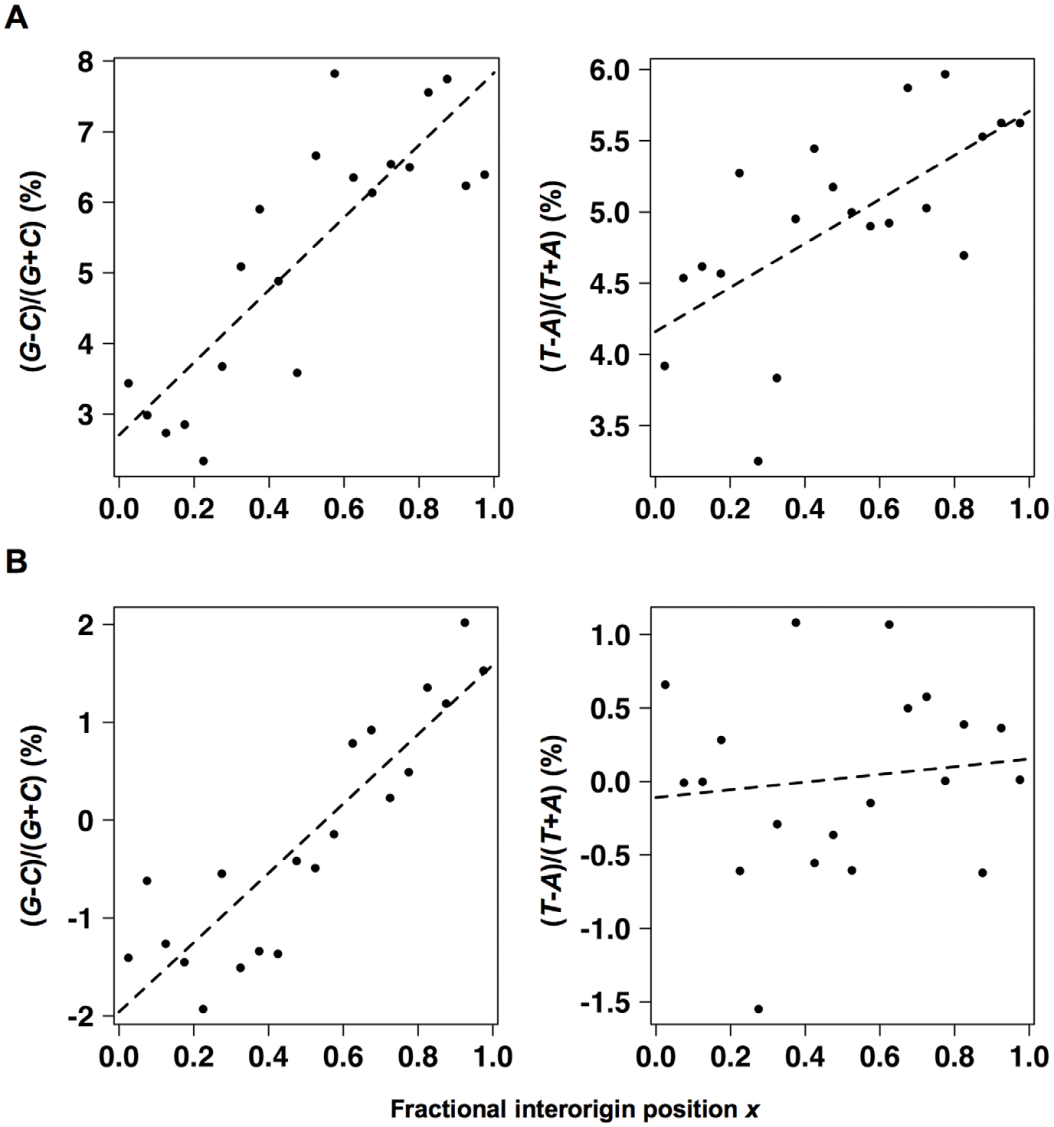
Variations of average GC and TA skews across interorigin intervals in *Candida albicans*. The skews, given in percent, were computed for third codon positions (A) and for intergenes (B). The lines correspond to the fits determined using quasibinomial models (see Materials and Methods).

*I_GC,TA_* represents the simplest way to combine *I_GC,cod_*, *I_TA,cod_*, *I_GC,int_* and *I_TA,int_*, but other combinations of these indices could be more powerful for discriminating origin-containing intergenes. Logistic regression analysis was performed to define the linear combination of *I_GC,cod_*, *I_TA,cod_*, *I_GC,int_* and *I_TA,int_* (computed for *L* = 20 kb) that describes best the relationship between asymmetry indices and the probability for an intergene to be used as an origin. The regression coefficients for *I_GC,cod_*, *I_TA,cod_*, *I_GC,int_* and *I_TA,int_* were similar (19±2, 18±3, 11±3 and 13±3, respectively), which shows that the unweighted sum of the indices is close to the optimal linear combination.

Origin-prediction strategies based on skew analysis are limited by the fact that they rely on traces of the past activity of replication origins to infer the location of presently active ones. Only sufficiently efficient origins whose activity has taken place during a sufficiently long period can be detected by these methods. False negatives can thus correspond to recent or relatively unefficient origins and false positives to ancient origins that are no longer active. Nevertheless, the strategy using *I_GC,TA_* in *S. cerevisiae* allows to define sets of intergenes significantly enriched in currently active replication origins.

*A priori*, *I_GC,TA_* could also be employed for predicting the location of termination sites. However, it is more difficult to estimate the strategy performance in this case as only 71 termination regions (out of probably ∼ 300 since their number must match the number of origins) have been identified, and only with a precision of 2-to-10 kb [33] so that, in contrast to origins, they cannot be unambiguously assigned to specific intergenes.

### Investigating the Possibility of Inferring Skew Direction from Analysis of Centromeric Sequences

Strategies for predicting replication origins through skew analysis imply to know the skew directions (which strand, leading or lagging, is enriched in G and which one is enriched in T). In prokaryotes, this information can be deduced from the preferential coorientation of replication and transcription (the leading strand has been generally observed to harbor more coding sequences than the lagging strand, *e.g.* [7]). However, the leading strand is not enriched in coding sequences in *S. cerevisiae* [19] and anyway the multiplicity of origins in eukaryotic genomes would make this inference difficult. Another possibility consists in the deduction of skew directions through analysis of a few experimentally-determined replication origins, which was the case for the human genome [17].

Recently, Berman and collaborators showed that the centromeres of the yeast *Candida albicans* are systematically associated with a replication origin that is the first to fire on its chromosome and that centromeric sequences exhibit skew jumps characteristic of efficient replication origins, which are conserved among related species [20]. This observation opened the possibility to determine skew direction simply by analyzing centromeric sequences, in the species where centromeres are associated with skew jumps (including several *Candida* species, *Lodderomyces elongisporus* and *Yarrowia lipolytica*, as determined by [20]). However, as shown in Figure 6, the variations of the average GC skew (and of the average TA skew, data not shown) around *C. albicans* centromeres and around 274 confirmed and active *S. cerevisiae* origins are markedly different. First, the amplitude of the GC skew jump around *C. albicans* centromeres is very large (74%) compared to the amplitude of the GC skew jump around *S. cerevisiae* origins (9%), which is close to the values usually observed for replication-related skew jumps in the human or in eubacterial genomes [6], [17]. Second, replication-related GC skew is expected to shift abruptly at an origin position and then either to remain constant up to the termination site (in eubacterial genomes, see [6]) or to decrease (or increase) slowly down (or up) to a position where it reaches its average value, and which corresponds to the average fork convergence point (in eukaryotes, see [17]). GC skew variations around *S. cerevisiae* origins match this pattern (Figure 6B): the GC skew decreases and increases, respectively, on the left and on the right sides, with distance from the origin and, in both cases, reaches its average value at a distance of ∼ 20 kb. This is in keeping with the fact that the average interorigin distance in *S. cerevisiae* is ∼ 40 kb, so that the average position of fork convergence points is expected to be located ∼ 20 kb away from origins. In contrast, the GC skew changes abruptly at *C. albicans* centromeres but goes back to its average value at a distance of ∼ 3 kb from the zero-intersection point (Figure 6A). If these variations were related to replication, that would imply that fork convergence points would be located on average ∼ 3 kb from the zero-intersection point on both sides of the centromeres. This would imply in turn the presence of two highly efficient replication origins, located ∼ 6 kb from the zero-intersection point, on both sides of the centromeres. However, no variations in GC skew comparable to the jump present at the centromeres can be observed in these regions. Accordingly, it seems unlikely that the GC skew variations around *C. albicans* centromeres are linked to replication.

I then sought to investigate the existence of replication-related skews in *C. albicans* by analyzing its replication timing program as determined in [20]. Origin positions were identified as peak positions of the replication profiles (see Materials and Methods) and variations of average GC and TA skews across interorigin intervals were determined as previously described [19]. Briefly, interorigin intervals were divided into 20 subintervals marked with fractional positions due to their variable lengths. For each subinterval, the data of all interorigin intervals were binned, which corresponds either to third codon positions (Figure 7A) or to intergenic sequences (Figure 7B), and average GC and TA skews were calculated. The GC skew increases significantly with interorigin position *x* both for third codon positions and for intergenes (*P* values equal to 4×10^−6^ and 2×10^−7^, respectively). In contrast, the TA skew increases significantly with increasing *x* for third codon positions, but not for intergenes (*P* values equal to 2×10^−3^ and 0.6, respectively). Similar results for third codon positions were obtained when the analysis was repeated excluding the 20% of the genes with the highest codon adaptation index (CAI) values, so as to eliminate the strongest expression-related effects on GC and TA skew variations (see Materials and Methods and Figure S1A, *P* values equal to 10^−7^ and to 2×10^−3^, respectively, for the correlations between *x* and the GC and TA skews). Finally, significant correlations between *x* and the GC and TA skews were also observed when the analysis was restricted to 4-fold degenerate codons (Figure S1, B and C, *P* values equal to 6×10^−4^ and to 7×10^−3^, respectively, when all genes were taken into account; *P* values equal to 5×10^−5^ and to 10^−3^, respectively, when excluding the 20% of the genes with the highest CAI values).

These results consistently indicate the presence of a replication-associated GC skew. The existence of a replication-related TA skew is also probable despite the lack of significant TA skew variation with interorigin position in intergenic sequences. The directions of these skews indicate that the leading strand is enriched in adenines and cytosines, as in *S. cerevisiae* and *K. lactis*, in contrast to the conclusion previously proposed [20]. Since the skew jumps associated with centromeric sequences in *C. albicans* are not linked to replication, analysis of centromeric sequences in related species cannot be used to infer the direction of replication-related skews.

### Conclusions

I propose here the definition of a global asymmetry index *I_GC,TA_* reflecting the difference in replication-related skews between the two sides of a given position in the genome. The value of this index depends on the past activity of neighboring replication origins and termination sites. Consequently *I_GC,TA_* can reflect some features of the current replication process only to the extent where they have remained unchanged since the time of skew formation (which may or may not extend up to now). Correlations were observed in *S. cerevisiae* between the location of the extrema of *I_GC,TA_* and the position of replication origins or termination sites, as well as between *I_GC,TA_* and origin efficiency. These correlations attest the stability of replication features in yeast and show that information relative to replication in present-day strains can be derived from the analysis of *I_GC,TA_*. In particular, asymmetry indices could be used for origin prediction in naive eukaryotic genomes, either alone or in combination with other approaches.

The use of asymmetry indices such as *I_GC,TA_* (which do not mix coding and template sequences) is well-adapted to genomes in which replication origins are confined to specific, interspersed sequences (like intergenes in yeast) and which contain a high proportion of coding sequences. In these cases indeed, the information derived from third codon positions is considerable and the measure of the difference in replication-associated skew between the two sides of a position is not distorted by the proportions of coding and template sequences. Generally speaking, a global asymmetry index can be defined for any genome as the unweighted sum of GC and TA asymmetry indices computed separately for different types of sequences under weak selective pressure (third codon positions, introns and intergenes). This global index must be computed knowing the directions of the GC and TA skews so as to properly add (or substract) the GC and TA skews and to reflect the probability of a sequence to be used as a replication origin. The knowledge of GC and TA skew directions is thus crucial to this approach and could be gained from the analysis of replication timing profiles or of a few experimentally-determined origins.

## Supporting Information

Figure S1 **Variations of average GC and TA skews for third codon positions across interorigin intervals in** ***Candida albicans*** **.** (A) All codons were taken into account, but the 20% of the genes with the highest CAI values were excluded from the analysis. (B) Only fourfold degenerate codons were analyzed. (C) Fourfold degenerate codons were analyzed, excluding the 20% of the genes with the highest CAI values. The lines correspond to the fits determined using quasibinomial models.
(TIF)
