# Supplementary figures and images for: Asymmetry Indices for Analysis and Prediction of Replication Origins in Eukaryotic Genomes
Source: PLoS One. 2012 Sep 27;7(9):e45050. doi: 10.1371/journal.pone.0045050 (PMC3459929; doi:10.1371/journal.pone.0045050)

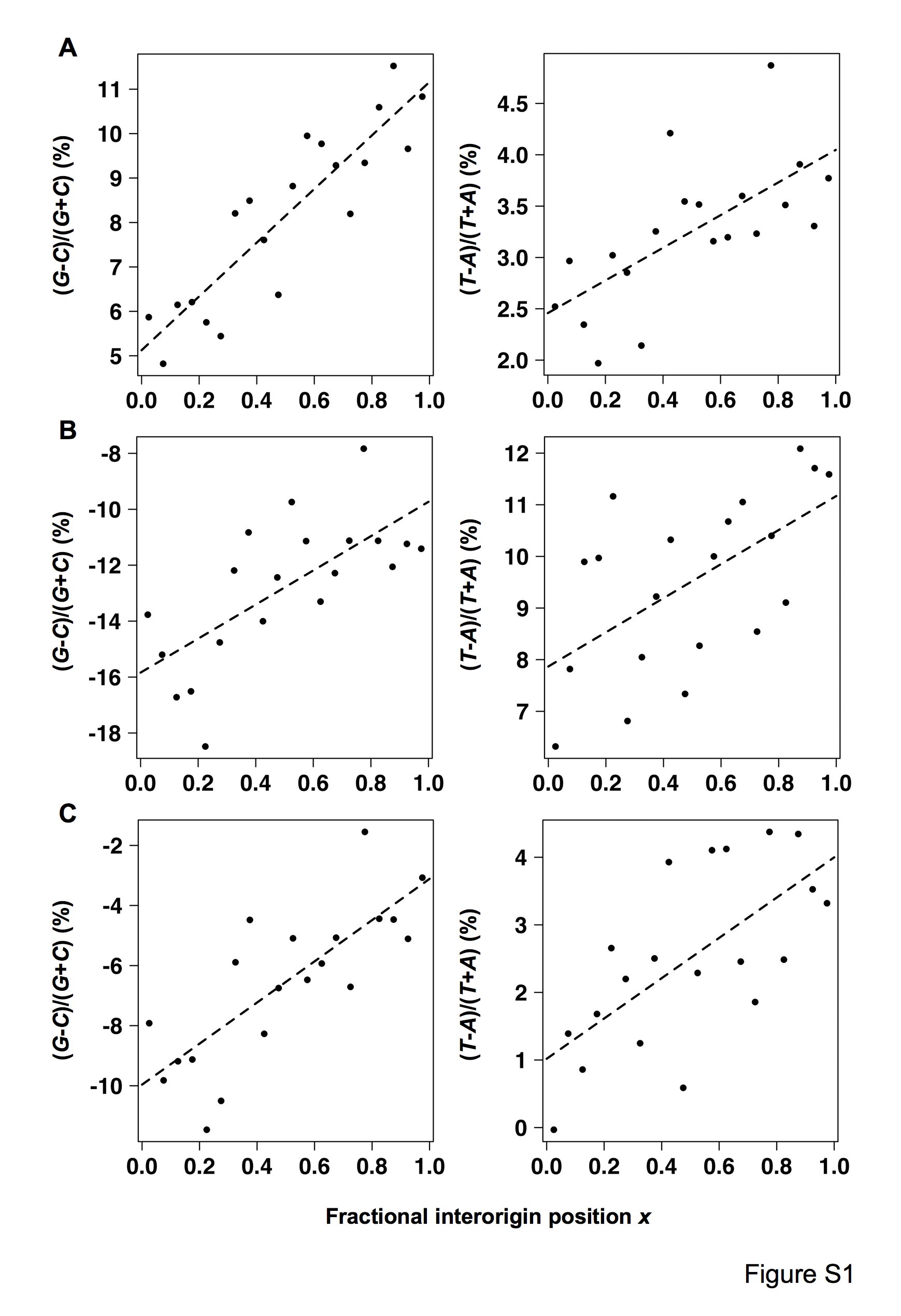

Supplement: Figure S1 — Variations of average GC and TA skews for third codon positions across interorigin intervals in Candida albicans . (A) All codons were taken into account, but the 20% of the genes with the highest CAI values were excluded from the analysis. (B) Only fourfold degenerate codons were analyzed. (C) Fourfold degenerate codons were analyzed, excluding the 20% of the genes with the highest CAI values. The lines correspond to the fits determined using quasibinomial models. (TIF) [file pone.0045050.s001.tif]
